# Automated data collection and real-time data analysis suite for serial synchrotron crystallography

**DOI:** 10.1107/S1600577518016570

**Published:** 2019-01-01

**Authors:** Shibom Basu, Jakub W. Kaminski, Ezequiel Panepucci, Chia-Ying Huang, Rangana Warshamanage, Meitian Wang, Justyna Aleksandra Wojdyla

**Affiliations:** a Swiss Light Source, Paul Scherrer Institute, 5232 Villigen PSI, Switzerland

**Keywords:** serial synchrotron crystallography, protein crystallography, online data analysis, data merging, data acquisition

## Abstract

At the Swiss Light Source macromolecular crystallography (MX) beamlines the collection of serial synchrotron crystallography (SSX) diffraction data is facilitated by the recent *DA+* data acquisition and analysis software developments. The *SSX suite* allows easy, efficient and high-throughput measurements on a large number of crystals. The fast continuous diffraction-based two-dimensional grid scan method allows initial location of microcrystals. The *CY+* *GUI* utility enables efficient assessment of a grid scan’s analysis output and subsequent collection of multiple wedges of data (so-called minisets) from automatically selected positions in a serial and automated way. The automated data processing (*adp*) routines adapted to the SSX data collection mode provide near real time analysis for data in both CBF and HDF5 formats. The automatic data merging (*adm*) is the latest extension of the *DA+* data analysis software routines. It utilizes the *sxdm* (SSX data merging) package, which provides automatic online scaling and merging of minisets and allows identification of a minisets subset resulting in the best quality of the final merged data. The results of both *adp* and *adm* are sent to the MX MongoDB database and displayed in the web-based tracker, which provides the user with on-the-fly feedback about the experiment.

## Abstract 2

Implementation of the complete synchrotron serial crystallography (SSX) suite for real-time data collection and processing available at the Swiss Light Source macromolecular beamlines is reported. A detailed description of the latest software extensions, *i.e.* the SSX user interface, as well as online data processing and merging routines, is also given.

## 1. Introduction   

The emergence of serial crystallography at the X-ray free-electron lasers (XFELs) and its subsequent adaptation at the synchrotron X-ray sources allowed macromolecular crystallography (MX) expansion towards previously inaccessible structural biology targets such as membrane proteins or large complexes, which tend to crystallize as micrometre-sized crystals. Serial femtosecond crystallography (SFX) was developed for free-electron laser facilities (Emma *et al.*, 2010 ▸), where each submicrometre crystal (≤1 µm) is shot only once with an extremely intense short pulse (∼40 fs) of coherent X-ray beam (Chapman *et al.*, 2011 ▸; Boutet *et al.*, 2012 ▸). Inspired by SFX, similar data collection strategies have recently been adopted at various synchrotron facilities (Gati *et al.*, 2014 ▸; Botha *et al.*, 2015 ▸; Nogly *et al.*, 2015 ▸). In the case of crystals delivered into the X-ray beam with injectors (Weierstall *et al.*, 2014 ▸; Botha *et al.*, 2015 ▸; Nogly *et al.*, 2015 ▸; Weinert *et al.*, 2017 ▸; Martin-Garcia *et al.*, 2017 ▸) only a couple of diffraction snapshots per crystal may be collected. Alternatively, crystals can be mounted on a solid support, such as a mesh loop or chip (Cohen *et al.*, 2014 ▸; Baxter *et al.*, 2016 ▸; Huang *et al.*, 2015 ▸; Meents *et al.*, 2017 ▸; Owen *et al.*, 2017 ▸), where small wedges of rotation data (so-called minisets) of typically 10–20° are collected. This advanced data collection strategy from fixed target microcrystals is generally known as serial synchrotron crystallography (SSX) (Diederichs & Wang, 2017 ▸). A recently developed *in meso in situ* serial crystallography (IMISX) plate is an alternative to the standard fixed-target technique, in which membrane proteins are crystallized in lipid cubic phase (LCP) and sandwiched between thin plastic windows (Huang *et al.*, 2015 ▸, 2016 ▸). The IMISX plate allows collection of SSX data from crystals in native condition at either cryogenic or room temperature without the need for crystal harvesting.

Third-generation low-emittance X-ray sources, which provide micrometre-sized beam and development of fast noise-free detectors, enabled collection of SSX from 5 to 30 µm crystals (Coulibaly *et al.*, 2007 ▸; Bowler *et al.*, 2016 ▸). In parallel with hardware improvements, the ongoing software developments allow more intuitive experiment control *via* dedicated GUIs (McPhillips *et al.*, 2002 ▸; Ueno *et al.*, 2005 ▸; Skinner *et al.*, 2006 ▸; Yamada *et al.*, 2008 ▸; Gabadinho *et al.*, 2010 ▸; Stepanov *et al.*, 2011 ▸; Winter & McAuley, 2011 ▸; Wojdyla *et al.*, 2018 ▸) and provide online data processing results (González *et al.*, 2008 ▸; Incardona *et al.*, 2009 ▸; Pothineni *et al.*, 2014 ▸; Monaco *et al.*, 2013 ▸; Winter, 2010 ▸; Vonrhein *et al.*, 2011 ▸; Tsai *et al.*, 2013 ▸). Recently, automatic data collection and analysis of the SSX data have been reported, where the *MeshAndCollect* GUI was developed and interfaced with *mxCUBE* (Zander *et al.*, 2015 ▸). Moreover, two other pipelines solely dedicated to processing and assembly of a complete high-quality dataset from multiple minisets have been reported (Guo *et al.*, 2018 ▸; Yamashita *et al.*, 2018 ▸).

Automation of data collection and analysis of the SSX measurements is a challenging task. Efficient selection of well diffracting microcrystals and automated collection of multiple minisets are crucial to enable high-throughput experiments. Subsequent data analysis demands an optimal usage of computing infrastructure in order to keep up with fast data collection (∼6 s/10–20° wedge). Standard data processing routines require adjustments of parameters to successfully adapt to small wedges of data. Moreover, results of data processing of individual minisets are often not adequate for users to interpret and assess the quality of crystals, as standard crystallographic metrics fail to provide useful statistics for extremely weak data. Real-time automatic merging procedures are necessary to provide users with appropriate tools to judge the quality of the collected SSX data on-the-fly. Fundamental to successful SSX data processing automation are (i) a reliable pipeline for the selection of minisets and (ii) software infrastructure which is able to monitor the progress of the SSX data collection and initialize on-the-fly merging at distinct intervals.

In order to extract interpretable statistics from a large amount of collected minisets a few aspects are important to consider, namely identification of experimental errors, elimination of poor quality non-isomorphous minisets, and precise selection of a cluster of highly correlated minisets. Each small wedge of data collected from micrometre-sized crystals is usually weak and suffers from both systematic and random errors. At the same time merging introduces systematic errors due to inherent non-isomorphism between any two datasets. Decoupling these two types of errors and discarding datasets with large systematic errors (non-isomorphous) is not trivial. Various concepts have been developed for crystallographic data selection, scaling and merging such as hierarchical cluster analysis (HCA) based on cross-correlation among datasets (Giordano *et al.*, 2012 ▸; Santoni *et al.*, 2017 ▸), unit-cell-based hierarchical clustering (BLEND; Foadi *et al.*, 2013 ▸), genetic algorithm (CODGAS; Zander *et al.*, 2016 ▸) and xscale_isocluster (Diederichs, 2017 ▸). All these concepts have been successfully demonstrated on various cases to provide complete and high-quality datasets by careful data selection and merging. However, identification of the non-isomorphism is still one of the main limitations of the existing algorithms. Therefore, minisets selection is an active field of research currently without a single robust fit-it-all metric.

Here we present the complete *SSX suite* for real-time data collection and processing available at the Swiss Light Source (SLS) MX beamlines. We describe the software components utilized at each consecutive step of the experiment, from identification of well diffracting microcrystals, *via* automated collection of minisets and online data analysis, to display of results in the web-based tracker and results storage in the dedicated database. The on-the-fly merging routine utilizes the in-house-developed SSX data merging (*sxdm*) package, which aims at identifying the best subset of minisets. Moreover, we demonstrate the performance of our *SSX suite* by collecting and fully processing 100 minisets from a membrane protein PepT_St_ within two hours of beam time.

## 2. Hardware and software infrastructure   

The in-house-developed *DA+* data acquisition (daq) and analysis software is a collection of distributed services and utilities (Wojdyla *et al.*, 2018 ▸). *DA+* relies heavily on a well maintained and reliable network, which allows efficient communication between beamline consoles, hardware components, data storage, computing clusters and database. The user defines an experiment in the graphical user interface (*DA+ GUI*), requests its execution (performed by the *DA+ server*) and inspects the results of the data processing in the web-based *adp-tracker* on the beamline user console.

The SLS Macromolecular Crystallography (MX) Group operates one bending-magnet (X06DA) and two undulator (X06SA and X10SA) beamlines. Each beamline is equipped with a computing cluster, which is used for online data processing. Beamlines X06DA and X10SA benefit from four Dual Xeon E5-2697v2 (2.70 GHz) 24 cores, 256 GB RAM, Scientific Linux 6.4 nodes. Installation of the EIGER X 16M detector (Dectris) at the X06SA beamline necessitated upgrade of the cluster to 16 Dual Xeon E5-2697v4 (2.30 GHz) 36 cores, 256 GB RAM, Scientific Linux 7.0 nodes. This extremely powerful cluster allows on-the-fly processing of large grid scans, as well as automatic data processing and merging of diffraction images written in NeXus data format (Könnecke *et al.*, 2015 ▸) within an HDF5 container (HDF5, 2014 ▸).

## 3. *SSX suite*   

The *SSX suite* is an extension of the *DA+* data acquisition and analysis software. It includes already existing software components which were expanded to enable an advanced SSX data collection strategy, as well as newly developed software utilities (*CY+ GUI*, *adm*, *sxdm*). The SSX data collection and subsequent analysis follow a well defined path, which includes the following steps and associated *DA+* software components:

(1) sample mounting and centring, accomplished in the *DA+ GUI*,

(2) identification of well diffracting microcrystals with fast grid scan in the *DA+ GUI*,

(3) evaluation of grid scan results and automated collection of multiple minisets performed in the *CY+ GUI* (Section 4),

(4) automatic data processing (*adp*) of individual minisets (Section 5),

(5) automatic data merging (*adm*) of multiple minisets using the *sxdm* package (Section 6),

(6) automatic display of *adp* and *adm* results for the on-the-fly inspection (*adp-tracker*) and storage in the database (*mxdb*) (Section 7).

The first two steps of the aforementioned procedure were described in detail previously (Wojdyla *et al.*, 2016 ▸, 2018 ▸), while the remaining steps (3)–(6) are discussed in the subsequent sections (as indicated above).

## 4. *CY+ GUI*   

**Figure 1:**
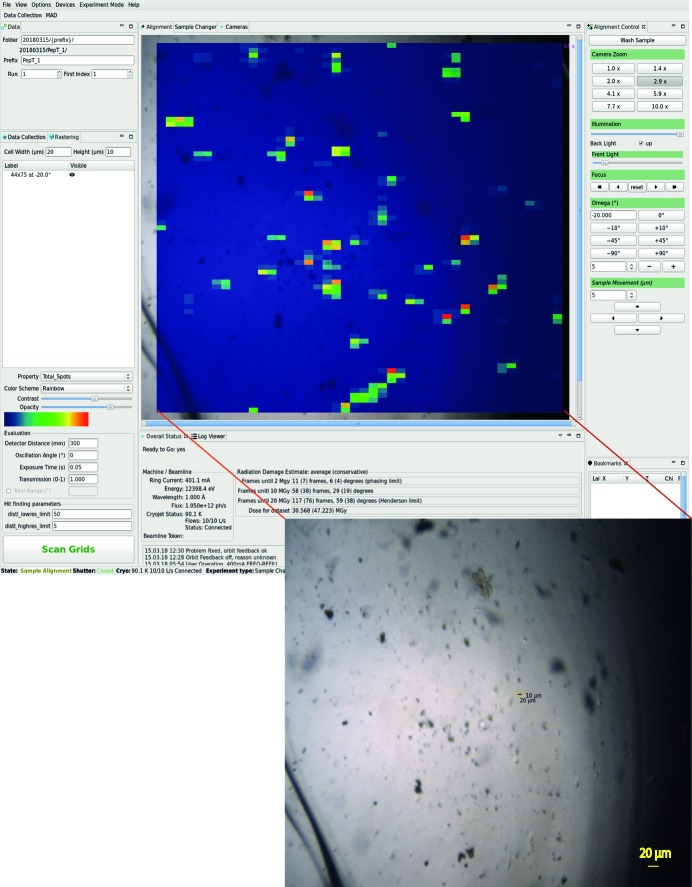
The *DA+ GUI* with heat map of diffraction results of a grid scan performed on the sample containing 10 µm × 10 µm × 15 µm PepT_St_ crystals (shown as inset at the bottom). The red colour indicates the highest number of diffraction spots.

In the first step of the SSX data collection procedure a sample support with multiple microcrystals is mounted onto the goniometer and centred in the *DA+ GUI* camera view. Subsequently, well diffracting microcrystals are identified with a fast continuous two-dimensional grid scan, which is defined and executed in the dedicated *DA+ GUI* ‘Rastering’ tab. Grid scan diffraction images are analysed with the labelit.distl package (Zhang *et al.*, 2006 ▸) and results are superposed onto the sample view in the *DA+ GUI* (Fig. 1 ▸). In the following steps, evaluation of the grid scan results and automated collection of multiple minisets are performed within the *CY+ GUI*.

**Figure 2:**
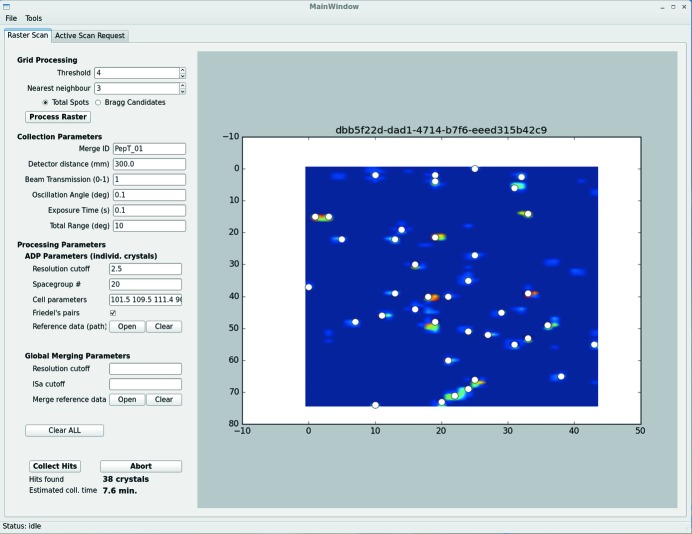
The *CY+ GUI* with results of the grid scan heat map from Fig. 1 ▸. White circles indicate 38 identified positions (hits), at which minisets will be automatically collected. The left-hand panel of the *CY+ GUI* main window contains three sections, which allow grid processing, data collection and processing parameters to be defined.

The *CY+ GUI* is written in Python 3.5 using PyQt4, which is a Python-binding of the open source cross-platform GUI Qt toolkit, and Qt Designer tool for designing and building of GUIs. The main *CY+ GUI* ‘Raster Scan’ window consists of a grid scan heat map, displayed on the right, and a left-hand panel with three sections (‘Grid Processing’, ‘Collection Parameters’ and ‘Processing Parameters’), which allow the number of processing and collection parameters to be defined (Fig. 2 ▸). After grid scan analysis for a given sample is inspected in the *DA+ GUI*, threshold and nearest-neighbour parameters are adjusted accordingly in the *CY+ GUI* ‘Grid processing’ tab and the grid scan heat map is loaded into the *CY+ GUI* by clicking the ‘Process Raster’ button. The ‘Grid processing’ tab allows grid scan output analysis based on labelit.distl results for total spots or Bragg candidates to identify well diffracting positions, where minisets will be collected. These so-called hits are shown in the grid scan heat map view as white dots. At the bottom of the *CY+ GUI* left panel the total number of found hits and estimated data collection time are displayed. Changing threshold and nearest-neighbour parameters allows optimization of the number of hits and length of data collection. It is also possible to add (shift and left mouse button click) or remove (ctrl and left mouse button click) hit points manually at a given position within the heat map with dedicated key combinations.

In the *CY+ GUI* ‘Collection parameters’ tab the user can input experimental data collection parameters (such as detector distance or miniset total angular range), as well as a mandatory Merge_ID. All minisets tagged with the same Merge_ID belong to the same project (regardless of the location of the diffraction data) and will be merged together in the *adm*. The ‘Processing parameters’ tab allows specifying ‘ADP parameters’ for processing of the individual minisets by the *adp* and ‘Global Merging Parameters’ used by the *adm* routines. All data processing parameter fields may be left empty, in which case they take default values.

Pressing the ‘Collect Hits’ button starts automatic and serial collection of minisets at each hit position specified in the *CY+ GUI*. At the same time automatic data processing (*adp*) and automatic data merging (*adm*) routines are triggered.

## 5. Automatic data processing of SSX data   

**Figure 3:**
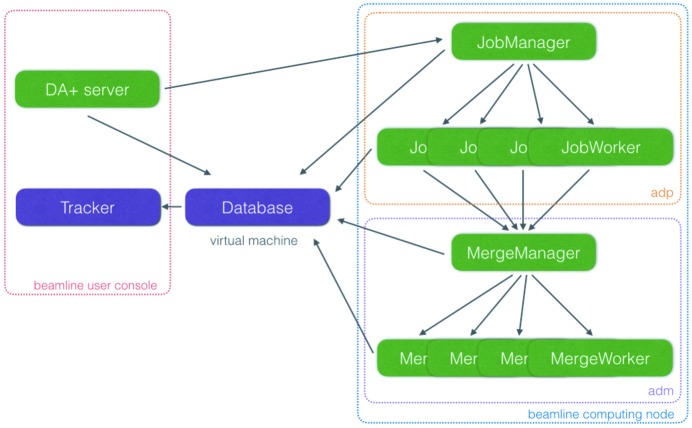
Schematic representation of distributed *DA+* daq and analysis software network. The beamline user console is delineated with a red dashed line and the beamline computing node with a blue dashed line. *Adp* and *adm* software utilities are outlined with orange and purple dashed lines, respectively.

Any data collection request, whether it is a standard dataset (*via* the *DA+ GUI*) or SSX minisets (*via* the *CY+ GUI*), is executed by the *DA+ server*. This central daq component communicates received requests to automatic data processing (*adp*) and the *mxdb* database (Fig. 3 ▸). Every data collection request received by the *DA+ server* corresponds to a single request sent to the *adp* regardless of whether it contains a single dataset (standard data collection), a few datasets (MAD or native-SAD) or multiple minisets (SSX). The *adp* was divided into two modules to allow optimal processing of not only standard datasets but also data from advanced collection protocols. The first *adp* module, called JobManager, receives a message from the *DA+ server*, analyses it and modifies its content. If necessary, JobManager splits the message into multiple messages, each representing a single dataset/miniset, which are forwarded to the second module. The second *adp* module consists of multiple instances of processes (Job­Workers) distributed throughout beamline-specific computing nodes which perform data processing and communicate results to the *mxdb* database. Moreover, in the case of data collection types, which require merging (such as native-SAD or SSX), the JobWorkers send merging request messages to the *adm* utility. The number of workers is adjusted based on the beamline’s computing power and data format (CBF or HDF5). For example, to keep up with the throughput of SSX data collection with an EIGER X 16M, 13 dedicated SSX and 4 standard dataset JobWorkers are distributed throughout the X06SA nodes. Each JobWorker processes a single SSX miniset using fast_xds, which is a Python wrapper for *XDS* (Kabsch, 2010 ▸). Processing parameters, such as resolution cut-off, space group, cell parameters, Friedel’s law and a reference dataset can be provided to the *adp* in the *CY+ GUI*.

## 6. Automatic merging of SSX data   

The automatic data merging (*adm*) utility performs online merging of SSX data. The main goals of the *adm* are to identify a subset of minisets resulting in the best quality of the final merged data (with the *sxdm* pipeline) and to provide users with the real-time feedback about the ongoing measurement. The *adm*, similarly to the *adp*, is divided into two main modules (Fig. 3 ▸). The MergeManager, the first *adm* module, receives a message from the *adp* and analyses its content. Currently, only SSX data can be merged by the *adm*; however, support for other experiments (such as native-SAD) was anticipated during software engineering. MergeManager internally counts all collected minisets for a given Merge_ID (specified in the *CY+ GUI*) and at predefined hardcoded intervals (10, 20, …, 100, 120, …, 200, 250, …, 800) sends a merging request to the second *adm* module. The second module, called MergeWorker, performs merging using the SSX scaling and merging (*sxdm*) package (details in Section 6.1) and sends *adm* results to the *mxdb* database.

### 6.1. SSX data scaling and merging utility   

The in-house-developed SSX data merging (*sxdm*) combines various concepts of data selection available in the literature (Foadi *et al.*, 2013 ▸; Giordano *et al.*, 2012 ▸) into one utility. Instead of deciding automatically which selection method is the most successful for a given project, the *sxdm* saves results from each step of the pipeline allowing the experimenter to assess all the outputs.

The *sxdm* is written as a standalone modular package, which is imported into the *adm* MergeWorker module. The *sxdm* requires the cctbx library and Python 2.7 environment, which includes numpy, scipy and matplotlib. Due to its modular nature, the package is easily expandable and sustainable, and in principle can be used to select and merge SSX data collected at any synchrotron beamline.

**Figure 4:**
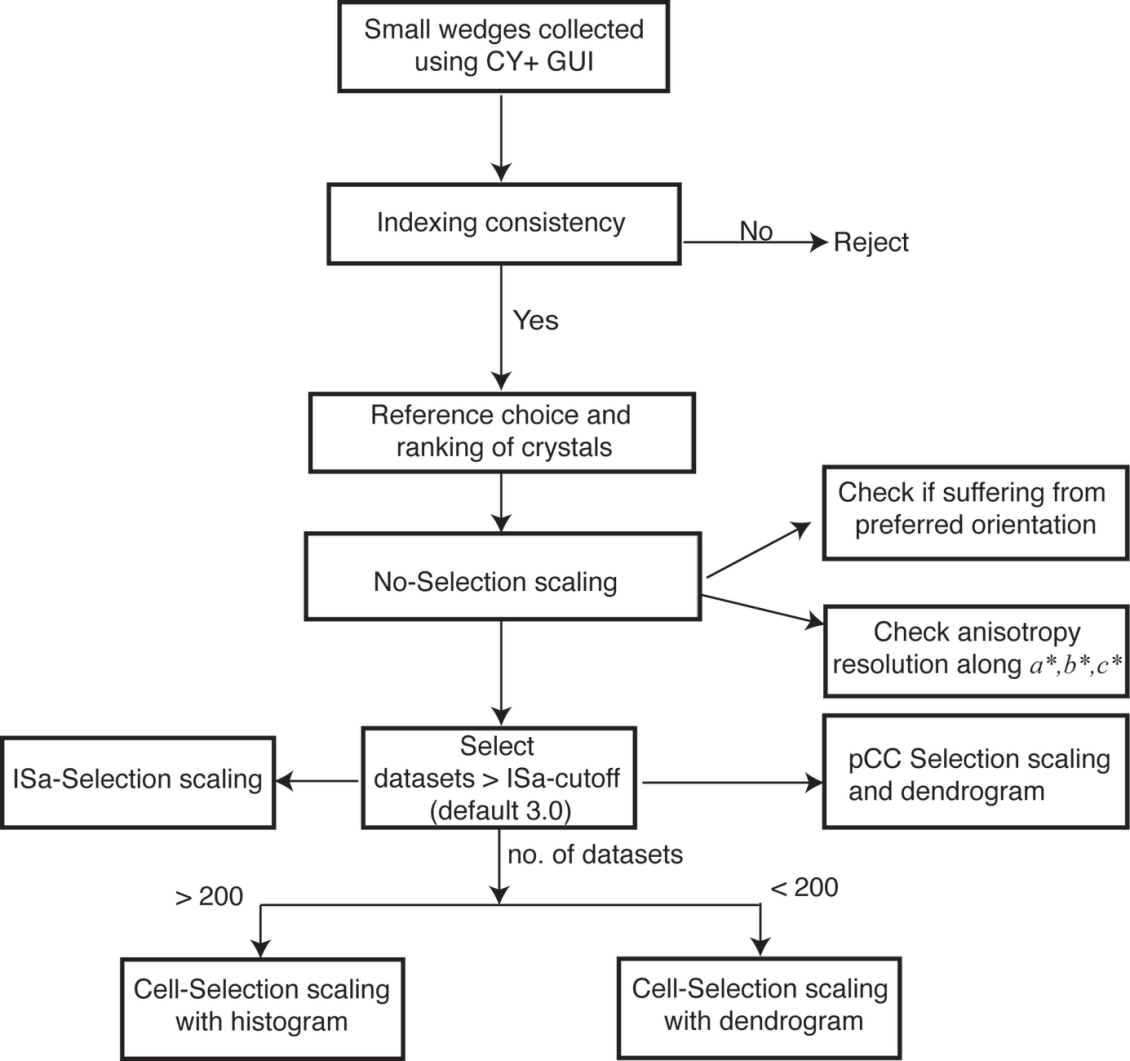
The *sxdm* work flowchart, showing steps for the online data selection and merging. Each step in the *sxdm* package is a callable object with instance methods. Users can perform each step independently in offline mode in any sequence.

The *sxdm* requires *XDS* output file XDS_ASCII.HKL and uses *XSCALE* at various stages of multiple minisets scaling. It also uses cctbx libraries for cross-correlation (CC) calculation and symmetry assessment between different datasets. The *sxdm* package provides a wrapper function, which calls the Merge_utls class to perform data selection and merging in the following steps (Fig. 4 ▸).

#### 6.1.1. Preparatory step   

The *sxdm* requires two mandatory arguments, which within *adm* are provided by the MergeWorker. The first argument is the list of *XDS* output files and the second is the type of data, *i.e.* SSX. Optional parameters, such as resolution cut-off, ISa cut-off and reference dataset for indexing check, can be provided in the *CY+ GUI*.

#### 6.1.2. Indexing consistency   

In this step the *sxdm* checks whether the unit-cell parameters of each miniset are within a 5% tolerance limit against the reference dataset (either specified in the *CY+ GUI* or the first dataset from the list of *XDS* files). Consistency between unit cell and space group assignment is also cross-checked and datasets which are indexed in a space group different from the reference dataset are rejected.

#### 6.1.3. Reference choice and ranking of crystals   

Once a subset of datasets is selected in the indexing consistency step, the dataset with the lowest Wilson *B*-factor is chosen as the reference dataset for the forthcoming scaling step. In addition, before the input file for *XSCALE* is prepared, all selected datasets are sorted in ascending order of mean *R*_meas_ values (calculated using the three lowest resolution shells). This sorting helps to identify a good reference dataset and improves relative scaling.

#### 6.1.4. No-selection scaling   

An initial round of *XSCALE* is performed on the *R*_meas_-sorted list of datasets. This step produces a scaled but unmerged reflection file (noSelect.HKL) which is the starting point for subsequent selection steps. No datasets are rejected at this stage.

#### 6.1.5. ISa-selection scaling   

ISa is an asymptotic *I*/σ(*I*) calculated by *XSCALE* as a product of two fitting parameters *a* and *b* in the error model (Diederichs, 2010 ▸). This error model is formed by fitting σ(*I*_*i*_) against the root-mean-square of (*I*_*i*_ − 〈*I*〉). *XSCALE* uses it to identify random and systematic errors associated with each dataset. Based on the first round of scaling, datasets with a very low ISa value (using an ISa cut-off either defined in the *CY+ GUI* or the default 3.0) are eliminated, which is followed by a second round of scaling (resulting in a ISa_Select.HKL file).

#### 6.1.6. Cell-selection scaling   

If the number of ISa-selected datasets is below 200, hierarchical clustering (scipy.cluster.hierarchy.linkage ward method) of the unit cell variation is applied and the clustering output is displayed as a dendrogram in the *adp-tracker*. Otherwise, unit-cell parameters are histogrammed in a Gaussian distribution and datasets which fall within 1.5σ (*i.e.* the width of distribution) are scaled using *XSCALE*. During the hierarchical clustering process, the entire ISa-selected list of datasets is described as an *n* × 6 matrix where each row stands for each dataset (where *n* equals the total number of datasets) and each unit cell is a function of six parameters (*i.e.* *a*, *b*, *c*, α, β, γ). Three resultant vectors are calculated on *ab*, *bc* and *ca* planes of the unit cell in three-dimensional space representing each unit cell as a data point of three variables or parameters in an (*x*, *y*, *z*) coordinate system [as descibed by Foadi *et al.* (2013 ▸)]. The three-dimensional Euclidean distance metric is calculated between any two such unit-cell resultant vectors and the most populated cluster is identified (RMS distance cutoff of 2). Scaling is performed on the datasets which belong to the most populated cluster (Cell_Select.HKL).

#### 6.1.7. pCC-Selection scaling   

In parallel to unit-cell clustering, another hierarchical clustering is performed based on pairwise cross correlation (pCC) values [described by Giordano *et al.* (2012 ▸) and Santoni *et al.* (2017 ▸)]. Correlations between symmetry-allowed common reflections from any two datasets are calculated with the cctbx library flex module. The mean CC calculated for low- and high-resolution shells may be deceptive and misrepresent true data quality either with very high values (a few common strong reflections) or with low values (too many common weak reflections). It is therefore calculated only for medium-resolution shell reflections (d_min = 4 Å and d_max = 8 Å). For *N* number of datasets, an *N* × *N* matrix is constructed, where diagonal elements are 1.0 (*i.e.* self correlation) and, since pCC[1,2] is equivalent to pCC[2,1], only a half-triangle of the *N* × *N* matrix is calculated to speed up the clustering process. This matrix is then passed to the hierarchical clustering method and clustered using ‘distance correlation’ as a metric (the scipy.cluster.hierarchy.linkage average method). Accordingly, a distance metric is used between any two correlation values in coordinate space to construct a dendrogram. Based on this dendrogram, datasets assigned to the most populated cluster are identified and scaled using a 0.8 distance correlation cutoff value (resulting in a pCC_Select.HKL file).

#### 6.1.8. Anisotropy and preferred orientation analysis   

In addition to data selection and merging, the *sxdm* package provides feedback about anisotropy and preferred orientation. In the case of SSX data collection from any type of fixed target (such as a chip), minisets can suffer from preferred orientation of crystals. This can be validated by plotting the distribution of multiplicity of each reflection (Huang *et al.*, 2016 ▸). A skewed or asymmetric distribution indicates a preferred orientation, otherwise a symmetric binomial distribution is expected. If a preferred orientation is detected, it is advisable to collect minisets at different orientations of the fixed target, while observing the plot in the *adp-tracker*. For anisotropy analysis the *sxdm* package utilizes *POINTLESS* and *AIMLESS* (Evans, 2006 ▸; Evans & Murshudov, 2013 ▸) to identify the resolution of scaled but unmerged datasets along a*, b* and c*. The output of anisotropy analysis is also displayed in the *adp-tracker* allowing the user to adjust the data collection strategy in real time.

## 7. *Mxdb* and *adp-tracker*   

The *adm* online processing relies on the database backend for the storage of metadata, results and processing parameters. The details of the *mxdb* database solution were described previously (Wojdyla *et al.*, 2018 ▸). For the purpose of integration with *adm*, (i) *mxdb* has been extended to store a new class of documents inserted by MergeManager and MergeWorkers, and (ii) a new API has been implemented to enable resilience and to track the status of internal variables of the online processing system.

The *DA+* daq and analysis software consists of a mesh of distributed utilities; therefore, an important factor of the ongoing development is ensuring that resilience protocols keeping the system consolidated in the unlikely event of failure are implemented. The current setup strongly depends on communication with appropriate timings; this means that any asynchronization caused for example by a network glitch could be detrimental to the *adp* and *adm* utilities. This task is delegated to the *mxdb* service, which, apart from archiving measurement metadata and processing results, also stores runtime variables of the *adm*, such as the current state of the merged datasets counter for a given project. In the case of an unexpected failure of *adm* caused by a crash or network instability, during initialization the last recorded status of *adm* is readily retrieved from the database and merging continues from where it had stopped. Conversely, the *adm* checks the status of the *mxdb* and is able to detect a crash of the database, in which case it raises the alarm and stops accepting new merging requests until the database is recovered. In such a way the system never ‘loses’ the miniset count during the down time and it recovers automatically without human intervention. Additionally, such an approach allows users to seamlessly continue the given project across different synchrotron visits.

**Figure 5:**
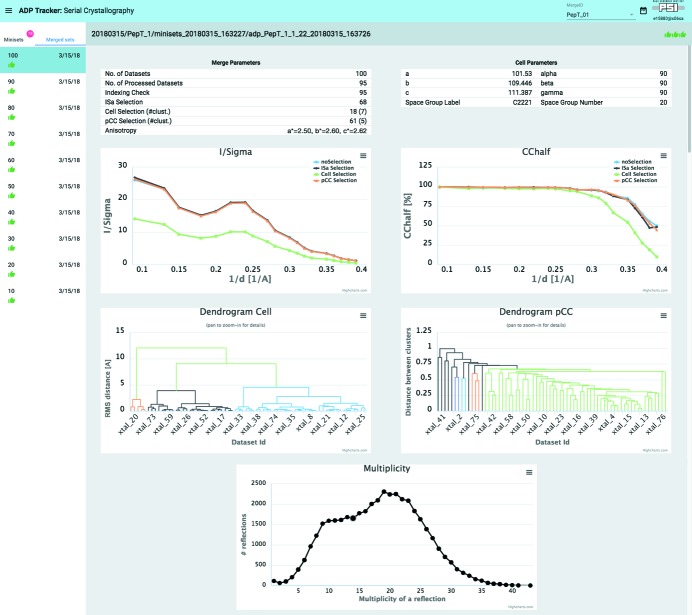
The *adp-tracker* ‘*SX-View*’ mode. The *adm* merging results for 100 PepT_St_ crystals are displayed. The ‘Cell Parameters’ table displays space group and cell parameters, while the ‘Merge Parameters’ table summarizes results of minisets selection from the *sxdm* package steps. *I*/σ and *CChalf* as a function of 1/*d* plots, *Cell* and *pCC dendrograms* with cluster distributions as well as a *Multiplicity* plot are shown.

To support the user during serial crystallography measurements and data merging, the *adp-tracker* web-based application [see details given by Wojdyla *et al.* (2018 ▸)] has also been extended to work with the *adm* online processing. It features a completely new mode of operation (*SX-View)* that enables users to monitor the SSX data processing output. By design its styling and user interface are identical to the ‘*Standard Data Collection Mode*’; however, it enables browsing between different Merge_IDs, tracks each single miniset that has been collected, and views the results of processing (Fig. 5 ▸). More importantly, it also gives meaningful insight into the quality of merged datasets by displaying the data in the form of plots and tables. All these features are implemented to help the user judge how the merging procedure is progressing and when the collected number of minisets is sufficient to consider the merged dataset complete. Currently five plots are presented to the user, namely *I*/σ and *CChalf* as function of *1/d, dendrograms* with cluster distributions and *Multiplicity* (Fig. 5 ▸). The plots are rendered using the Highcharts.js (Highcharts, 2018 ▸) library that integrates well with the Polymer 2.0 Javascript framework (Polymer, 2018 ▸) used to write *adp-tracker*. As described in more detail in previous work (Wojdyla *et al.*, 2018 ▸), *adp-tracker* is a web-browser event-driven application which always displays the current state of online processing without the need to refresh the page. The same holds true for the *SX-View* mode of *adp-tracker* and all the plots are updated in real time as the user progresses with data collection.

## 8. PepT_St_ showcase   

The PepT_St_ (peptide transporter from *Streptococcus thermophilus*) was crystallized (Lyons *et al.*, 2014 ▸) by *in meso* methods in an IMISX plate as described previously (Huang *et al.*, 2015 ▸). IMISX wells with boluses containing crystals were cut out from the plate, mounted onto a pin with Y-support on a magnetic base and flash-frozen in the liquid nitro­gen (Huang *et al.*, 2016 ▸). SSX data collection was performed at 100 K at the beamline X06SA using the EIGER X 16M detector at 12.4 keV photon energy with a beam size of 20 µm × 10 µm and a photon flux of 1 × 10^12^ photons s^−1^. A representative PepT_St_ sample, which was covered with a 44 × 75 grid and collected at 20 Hz (20 µm per frame) in 187 s, is shown in Fig. 1 ▸ (inset).

A heat map of the grid scan diffraction results superposed onto the sample and 38 hits (well diffracting microcrystals) identified based on the evaluation of grid scan results are shown in the *DA+ GUI* (Fig. 1 ▸) and *CY+ GUI* (Fig. 2 ▸), respectively. In total, one-hundred 10° minisets were collected (300 mm detector distance, 0.1° oscillation angle, 0.1 s exposure time and 100% transmission) from a single crystal-laden LCP bolus within two hours. Data were processed automatically by the *adp* and *adm* and output was inspected in the *adp-tracker* (Fig. 5 ▸).

Out of 100 collected minisets, 95 were processed and accepted in the ‘Indexing consistency’ step (space group *C*222_1_). Nearly 30 minisets were rejected based on the default 3.0 *ISa* cut-off, leading to 68 minisets scaled in the ‘ISa-selection’ step. Subsequent ‘Cell Selection’ and ‘pCC Selection’ steps selected 18 and 61 minisets, respectively. Both *I*/σ and *CChalf* as a function of 1/*d* plots clearly indicate that the scaling of the minisets selected at the ‘pCC Selection’ step resulted in a high-quality complete dataset at 2.6 Å resolution (highest resolution shell 1.22*I*/σ and 44.6% *CChalf*). The *pCC dendrogram* with cluster distribution indicates five clusters with the most populated one including 61 crystals. The multiplicity plot shows a distorted binomial distribution indicating a slightly preferential orientation of crystals; however, this is expected for protein crystals on a fixed-target type of support. In this case, the slight preferential orientation of the PepT_St_ crystals does not affect the completeness of the final ‘pCC Selection’ based dataset, which is nearly 100% (highest-resolution shell 100% completeness). Moreover, anisotropy analysis clearly indicates nearly isotropic diffraction of PepT_St_ crystals.

## 9. Summary   

The MX software team at the SLS have developed a suite for fast microcrystals identification, automatic SSX data collection, processing, minisets selection and merging. The *SSX suite* was developed as a modular extension to the already existing distributed *DA+* data acquisition and analysis software. The combination of already existing hardware and software solutions with newly developed software enables efficient collection of high-quality SSX data. The *SSX suite* is widely used by both academic and industry users measuring at the SLS X06SA and X10SA beamlines.

The in-house-developed *sxdm* package utilizes and organizes various scaling and merging concepts in a logical workflow providing users with instant feedback about the quality of collected minisets. Many concepts of the data selection procedures have been developed to date; however, none of them is sufficiently robust to be used standalone, making automatic decisions about selected subset of minisets a significant challenge. Outputs of each merging step utilized within the *sxdm* package are displayed in the web-based *adp-tracker* and saved in an easily interpretable fashion. Users are encouraged to browse results of the automatic routines and make educated on-the-fly decisions about further SSX data collection strategies.

The *CY+ GUI* consolidates many important aspects of the SSX data collection and processing into one software utility. It allows for the selection of well diffracting minisets based on the results of a fast grid scan (from *DA+ GUI*), as well as definition of data collection, *adp* and *adm* parameters. The cascade of events, which include automatic minisets collection, processing, merging, storage of results in the *mxdb* database and display in the *adp-tracker*, is initialized with a single click of a button. Our *SSX suite* enables highly automated, user-friendly and efficient experiments on a large number of crystals and allows a complete dataset to be obtained from one project within a few hours of beam time.
